# Theoretical Studies on the Structure and Intramolecular Interactions of Fagopyrins—Natural Photosensitizers of *Fagopyrum*

**DOI:** 10.3390/molecules27123689

**Published:** 2022-06-08

**Authors:** Sebastian Szymański, Irena Majerz

**Affiliations:** Faculty of Pharmacy, Wroclaw Medical University, Borowska 211a, 50-556 Wroclaw, Poland; irena.majerz@umw.edu.pl

**Keywords:** fagopyrins, conformation, hydrogen bond, QTAIM, NCI

## Abstract

**Simple Summary:**

The study determines the spatial structure and intramolecular interactions of fagopyrins—natural photosensitizers of *Fagopyrum* species. In silico calculations show many fagopyrin conformers characterized by the formation of strong intramolecular interactions.

**Abstract:**

Compounds characterized by a double-anthrone moiety are found in many plant species. One of them are fagopyrins—naturally occurring photosensitizers of *Fagopyrum*. The photosensitizing properties of fagopyrins are related to the selective absorption of light, which is a direct result of their spatial and electronic structure and many intramolecular interactions. The nature of the interactions varies in different parts of the molecule. The aim of this study is to determine the structure and intramolecular interactions of fagopyrin molecules. For this purpose, in silico calculations were used to perform geometry optimization in the gas phase. QTAIM and NCI analysis suggest the formation of the possible conformers in the fagopyrin molecules. The presence of a strong OHO hydrogen bond was shown in the anthrone moiety of fagopyrin. The minimum energy difference for selected conformers of fagopyrins was 1.1 kcal∙mol^−1^, which suggested that the fagopyrin structure may exist in a different conformation in plant material. Similar interactions were observed in previously studied structures of hypericin and sennidin; however, only fagopyrin showed the possibility of brake the strong OHO hydrogen bond in favor of forming a new OHN hydrogen bond.

## 1. Introduction

Fagopyrins are a group of compounds of natural origin found in plants of the genus Fagopyrum. Parts of these plants are commonly consumed by humans and animals throughout the world [1]. There are many species of Fagopyrum; however, the most consumed and studied are *Fagopyrum esculentum*, *F. tataricum*, and *F. cymosum* [2,3]. Parts of these plants provide a low-calorie, gluten-free food and a source of many elements and organic compounds of biological interest, such as rutin, quercetin, and fagopyrins [4,5,6,7,8].

Fagopyrins are anthraquinone derivatives characterized by a polycyclic system, which is interesting from the chemical point of view. The structure and intramolecular interactions of polycyclic compounds affect the physical and chemical properties and can influence potential applications of the compounds in pharmacy and medicine [9,10,11,12]. As shown in earlier work on hypericin [13] and sennidines [14], a highly substituted polycyclic system can exhibit a non-planar structure due to a variety of intramolecular interactions. At the time of writing this paper, data unambiguously define the spatial and electronic structures of fagopyrins. An explanation of this may be found in difficult and multi-stage processes needed to obtain the pure substance from plant material [15]. Additionally, the existence of unstable protofagopyrins [16,17] and the possibility of the existence of many derivatives [17,18] can be a problem.

The interest in fagopyrins is mainly due to their spectroscopic properties. They exhibit absorption of electromagnetic radiation in the wavelength range of the light around λ_max_ 550 and 590 nm [15,19,20]. Upon excitation, they are able to transfer energy to the oxygen molecule, thereby producing reactive oxygen species (ROS) [21]. Singlet oxygen and other ROS are responsible for cell damage. Easy light activation of fagopyrin molecules shows the potential to be used in photodynamic therapy [20]. On the other hand, high consumption of the *Fagopyrum* plants can lead to the light sensitivity in animals, called fagopyrism [22]. Additionally, there are reports of the possible hepatotoxic effect of consumption of food rich in *Fagopyrum* plants on dogs [23]. Leaving aside the potential dangers of the photosensitizing properties of fagopyrins, their potential for pharmacological use appears to be high. Easy excitation with energy from the visible range, confirmed antifungal [24] and antimicrobial [25] properties, and natural origin are promising for use in targeted photodynamic therapy.

Although general studies on the structure of fagopyrins were undertaken [17], no crystal structures are available, given the current state of knowledge. Additionally, such a strongly substituted double anthrone moiety has many possibilities for intramolecular interactions. The introduction of piperidine and pyrrolidine substituents in the hypericin molecule that, in fact, forms fagopyrin allows for the appearance of new intramolecular interactions.

Considering the intramolecular interactions occurring in fagopyrins, the OHO hydrogen bond system formed by hydroxyl groups bound to carbonyl oxygen is the most characteristic. As the strength of the hydrogen bond is determined primarily by the electronegativity of the atoms with which the proton is bound [26], the hydrogen bond of the OHO type is the strongest. The strength of the hydrogen bond is expressed in changes in geometry, consisting in shortening the distance between the donor and the acceptor of the proton and the location of the proton close to the center of the distance between the donor and the acceptor [27]. The OHO hydrogen bond system in fagopyrin is additionally strengthened by the participation of the hydrogen bonds in closed bond cycles in which double and aromatic bonds are present [28]. However, in the case of fagopyrins containing the piperidine and pyrrolidine substituents with the nitrogen atom and the possibility of conformational changes of the molecules, the presence of weaker hydrogen bonds of OHN type should be taken into account.

Therefore, it seems necessary to investigate the possible intramolecular interactions and structure of fagopyrins and compare them with the present state of knowledge using the example of hypericin.

The purpose of this paper is to use in silico methods to determine the molecular structure and interactions of still unknown fagopyrin molecules. Conformational analysis is carried out to determine the probable spatial and electronic structure of fagopyrins and, importantly, the intramolecular interactions of substituents that determine the pharmacological properties of these natural compounds. The results calculated for the gas phase will provide valuable knowledge about these interesting derivatives and will contribute to the future exploration of the pharmacological properties of fagopyrins. The conformational analysis will determine the minimum energy structure and possible formation of the double-anthrone system in plant material.

## 2. Materials and Methods

Determination of the spatial structure and conformational analysis of fagopyrin molecules were carried out using the Gaussian16 package [29]. Calculations were carried out using DFT/B3LYP/6-311++G(d,p) model with Grimme dispersion [30]. The optimized structures correspond to a minimum on local potential energy surfaces. QTAIM analysis was performed using the AIMALL program [31]. NCI analyses [32] were performed using the Multiwfn program [33]. NCI graphics were printed using the VMD program [34]. UV-VIS spectra and orbital analysis were performed with the ADF program [35].

## 3. Results and Discussion

**Scheme 1:**
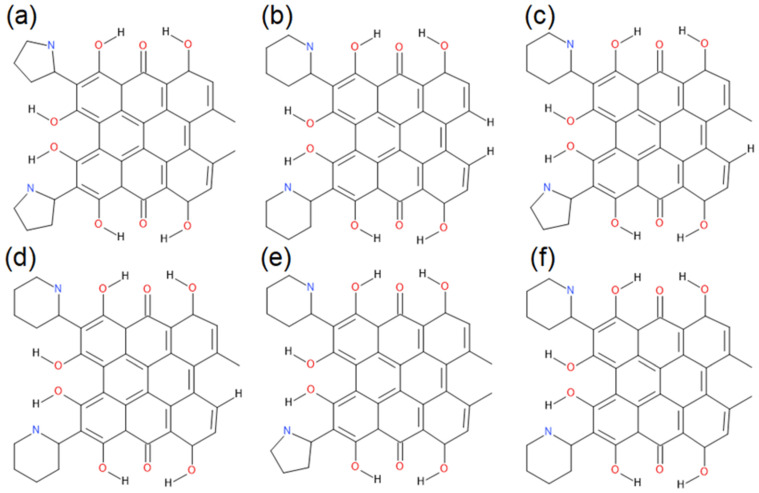
Analyzed structures of (**a**) fagopyrin A, (**b**) fagopyrin B, (**c**) fagopyrin C, (**d**) fagopyrin D, (**e**) fagopyrin E, and (**f**) fagopyrin F.

Six structures of fagopyrins A–F (Scheme 1) proposed by Benković et al. [17] were optimized in the gas phase. Conformational analysis was performed for the A–F structures by searching for the minimum on local potential energy surfaces.

Conformers representing different arrangements and interactions of substituent hydroxyl groups, carbonyl oxygen, piperidine, and pyrrolidine rings were compiled based on a structure with minimum energy. The close position of the substituents allowed for the formation of intramolecular interactions at the “peri” (O-H∙∙∙O∙∙∙H-O) and “bay” region of the molecules (O-H∙∙∙O-H). Additionally, the presence of piperidine and pyrrolidine substituents allowed for the formation of new interactions with the nitrogen atom.

### 3.1. Conformational Analysis of Fagopyrins

**Scheme 2:**
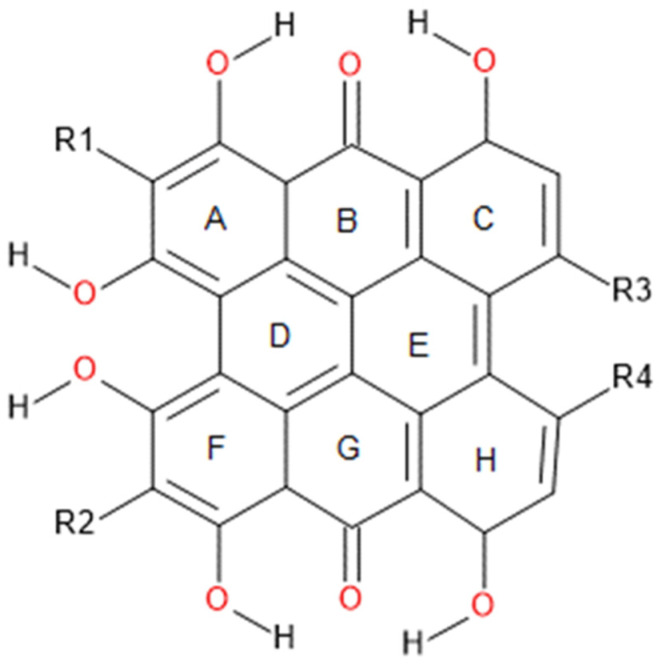
Double anthrone polycyclic system of fagopyrin A–H.

The structure of fagopyrin consists of a polycyclic system of eight rings. As shown in Scheme 2, each ring, named A–H, consists of six carbon atoms. Rings A–H are characterized by the presence of at least one substituent. In the A + B + C and F + G + H regions of the molecule, there are two hydroxyl groups and carbonyl oxygen. Such a close position of the substituents allows the formation of a hydrogen bond system in which hydroxyl groups are directed to the centrally located carbonyl oxygen. As shown for the hypericin molecule [13], such formation of strong hydrogen bonds is energetically preferred, and these hydrogen bonds are difficult to break. What is new in the structure of fagopyrin is the close position of piperidine and pyrrolidine substituents. These substituents can occur at positions named R1 and R2 (Scheme 2). The rings containing the substituents with nitrogen atoms give an additional possibility to form of intramolecular OHN hydrogen bond and possible breaking of the strong OHO hydrogen bonds. So far, no studies have been found on the arrangement of these substituents in fagopyrin molecules. Another interesting part of the fagopyrin molecule is the “bay” region consisting of the A + D + F ring system. In hypericin molecule, the preferred arrangement of the substituents in the “bay” region is to form an OHO hydrogen bond between two hydroxyl groups. In the fagopyrin molecule, the addition of piperidine and pyrrolidine rings allows the interactions to be directed to the nitrogen atom forming new OHN interactions. Another part of fagopyrin molecule that may affect the overall structure is the presence of R3 and R4 substituents. Depending on the type of fagopyrin A–F molecule, these parts can be substituted by protons or methyl groups. As shown for hypericin, the close distance of two methyl groups may cause strain in the entire molecule and can strongly affect the planarity of the polycyclic system. Such a variety of substituents and possible strain effects from methyl groups make the structure and intramolecular interactions in fagopyrin molecules worth describing.

**Figure 1:**
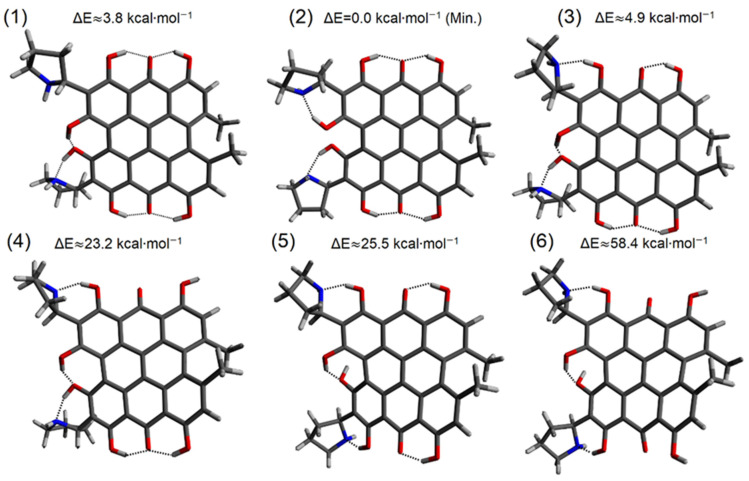
Conformers (**1**–**6**) of fagopyrin A.

Six low-energy conformers of fagopyrin A have been obtained, and structure 2 is the minimum energy conformer (Figure 1). The hydrogen bonds in the “peri” region of the molecule show alignment to the carbonyl oxygen. Two OHN hydrogen bonds in the “bay” region are preferred; however, breaking one OHN hydrogen bond in the “bay” region and forming OHO hydrogen bond between hydroxyl groups results in a total energy change of only 3.8 kcal∙mol^−1^ (structure 1). A similar change in the total energy of the system (ΔE ≈ 4.9 kcal∙mol^−1^) is caused by breaking a strong OHO hydrogen bond in the “peri” region and the formation of an OHN bond with the pyrrolidine substituent (structure 3). Breaking the OHO hydrogen bond in the “peri” region without the formation of another interaction destabilizes the fagopyrin structure and raises its energy (structure 4, 6).

**Figure 2:**
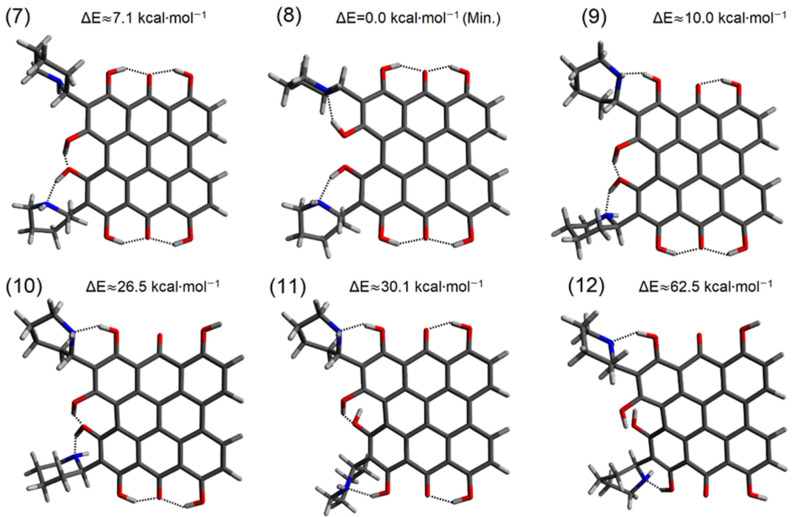
Conformers (**7**–**12**) of fagopyrin B.

For fagopyrin B, six low-energy conformers (Figure 2) have been obtained. Structure 8 showing the lowest energy is characterized by the OHO hydrogen bond arrangement in the “peri” region typical for anthrones. In the “bay” region, the OHN hydrogen bonds linking the hydroxyl group and the nitrogen atom are formed. The energy differences between the structures 7, 8, and 9 show the energy difference up to 10.0 kcal∙mol^−1^. The energy difference for these conformers is larger than the analogous difference for structures 1, 2, and 3 of fagopyrin A. Formation of OHN hydrogen bonds with the piperidine ring (fagopyrin A) shows larger energy differences than the formation of OHN interactions with the pyrrolidine ring (fagopyrin B). Additionally, it can be seen that the piperidine ring in the fagopyrin B prefers a “chair” conformation; however, interaction with the hydroxyl substituent in the “peri” and “bay” region can disrupt the chair conformation (structure 7–12). The presence of a free hydroxyl group (structure 10, 12) results in a significant increase in the energy of fagopyrin B. In contrast, the lack of the methyl groups brings the double anthrone system closer to planarity.

**Figure 3:**
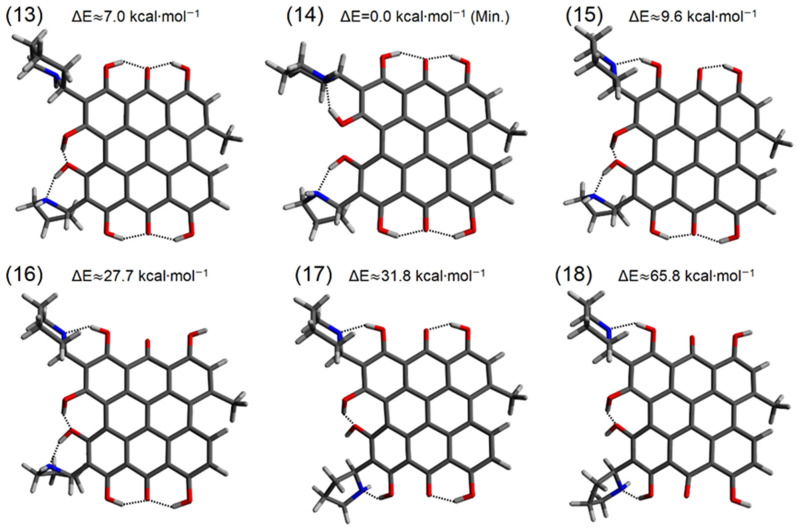
Conformers (**13**–**18**) of fagopyrin C.

Six conformers that were obtained for fagopyrin C (Figure 3) are characterized by low energy. The structure with the lowest energy (structure 14) favors the formation of OHN hydrogen bonds and the breaking of the OHO hydrogen bonds in the “bay” region of the molecule. The piperidine ring shows a “chair” conformation for all the obtained structures (structure 13–18). Breaking of the OHN hydrogen bond located in the “bay” region results in leaving the piperidine ring free and increasing the energy of the molecule by 7.0 kcal∙mol^−1^ (structure 13). Breaking of the OHO hydrogen bond in the “bay” region together with the formation of the OHN hydrogen bond with a hydroxyl group located in the “peri” region (structure 15) is associated with an energy increase of 9.6 kcal∙mol^−1^. Leaving the “free” hydroxyl group in the “peri” region results in a significant increase in the energy ΔE ≈ 27.7 kcal∙mol^−1^ (structure 16) and ΔE ≈ 65.8 kcal∙mol^−1^ (structure 18).

**Figure 4:**
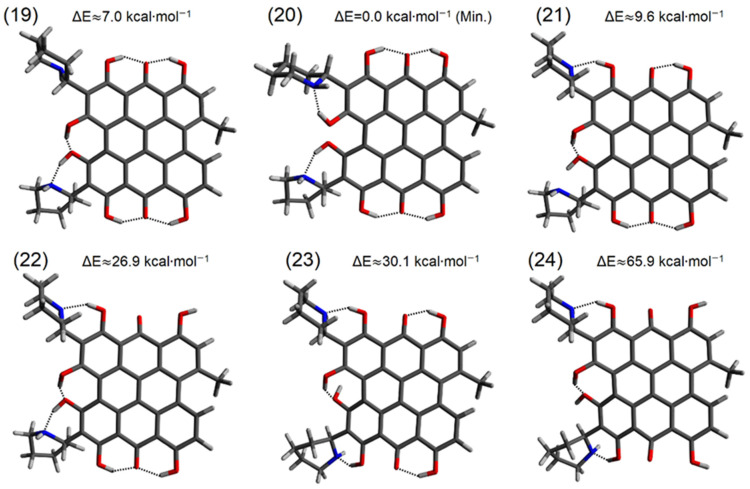
Conformers (**19**–**24**) of fagopyrin D.

Six low-energy conformers (Figure 4) were obtained for fagopyrin D. The lowest energy structure (structure 20) is characterized by the formation of an OHN hydrogen bond in the “bay” region. Structure 19 is characterized by a “hypericin-like” arrangement of the hydroxyl groups in the “bay”, and the “peri” region differs in energy by 7.0 kcal∙mol^−1^ from the lowest energy structure. The “chair” conformation is preferred for both piperidine rings in fagopyrin D. Structure 21 is characterized by the breaking of the strong OHO hydrogen bond in the “peri” region and the formation of an OHN hydrogen bond to the piperidine ring. Such transfer of the hydrogen interaction results in the energy difference of 9.6 kcal∙mol^−1^ to the minimum energy structure (structure 20). As in the fagopyrin A–C structure, the “free” hydroxyl group (22, 24) increases the energy of the conformer; however, in such a polycyclic system, this may not be a direct expression of breaking the OHN hydrogen bond but also due to possible structural changes of the multi-ring molecule. The formation of the OHN hydrogen bond in the “peri” region stabilizes the fagopyrin D molecule.

**Figure 5:**
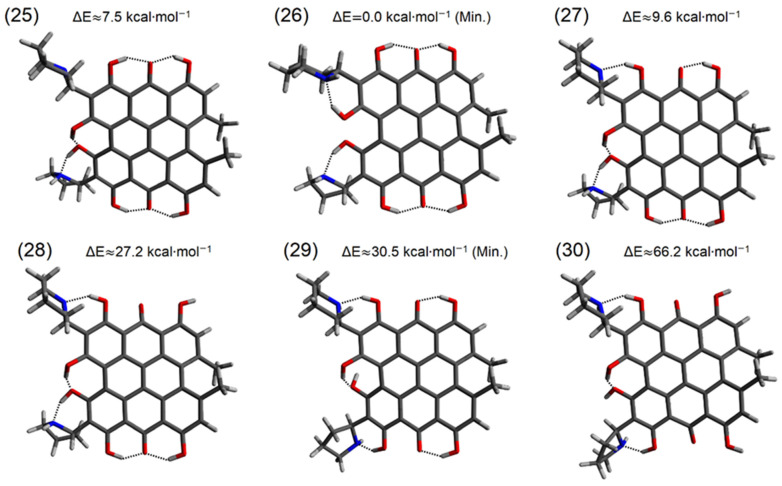
Conformers (**25**–**30**) of fagopyrin E.

Six conformers (Figure 5) were obtained for fagopyrin E. The lowest-energy conformer (structure 26) shows hydrogen bonding in the “bay” region of the molecule. The OHN hydrogen bonds are formed by the hydroxyl groups to both nitrogen atoms in the piperidine and pyrrolidine substituent. In the minimum-energy conformer, the hydrogen bonds in the “peri” region are directed to the carbonyl oxygen. The “chair” conformation of the piperidine substituent is preferred. Conformer characterized by the “free” piperidine group (structure 25) differs in the energy of 7.5 kcal∙mol^−1^. Additionally, breaking of OHO hydrogen bond in the “peri” region and transferring it to the “free” piperidine substituent (structure 27) raises the energy relative to conformer 25 by 2.1 kcal∙mol^−1^. As in the fagopyrin structures described previously, breaking of a strong OHO hydrogen bond in the “peri” region and leaving the hydroxyl group unbound raises the total energy of the polycyclic system (structure 28 and structure 30).

For fagopyrin F, six low-energy conformers were obtained. The lowest-energy structure again is characterized by the formation of the OHN hydrogen bonds in the “bay” region (structure 32). The chair conformation of the piperidine substituents is preferred. The energetically similar conformers 31 and 33 are characterized by an energy difference of 7.5 and 9.7 kcal∙mol^−1^, relatively to the minimum. As in conformers of fagopyrin E, it is possible to break the OHN hydrogen bond in the “bay” region and form an OHN hydrogen bond in the “peri” region. Breaking of the strong OHO hydrogen bond system in the “peri” region causes the deformation of the polycyclic system and deviates the molecule from planarity (36).

In general, the structure of fagopyrin tends to form OHN hydrogen bonds in the “bay” region. Energetically preferred formation of strong OHO hydrogen bonds to carbonyl oxygen in the “peri” region is evident in most conformers, and breaking of these interactions has the consequence of raising the energy of the system. Nevertheless, it is possible to break the strong OHO hydrogen bonds in the “peri” region in favor of the formation of an OHN hydrogen bond with the piperidine or pyrrolidine substituent. In summary, the introduction of piperidine and pyrrolidine substituents into the hypericin system provides an opportunity to form an OHN hydrogen bond instead of the strongest OHO.

### 3.2. Analysis of Geometry of Fagopyrin Structures

A parameter that describes the geometry of the fagopyrin conformers is the angle between the planes formed by the peripheral rings A–C, F–H, A–F, and C–H (Scheme 2). For hypericin, (Table 1) these angles are of degrees: A–C: 13.334, F–H: 12.363, A–F: 23.188, and C–H: 30.095. Selected conformers of fagopyrin A–F show significant similarity to the structure of hypericin. These conformers are 1, 7, 13, 19, 25, and 31. These conformers are characterized by different substitutions at the R1–R4 position but the hydroxyl groups in the “bay” and “peri” regions are oriented as in the hypericin molecule and form the same type OHO hydrogen bonds. The difference in the angle between the ring plane is the greatest for fagopyrin C and D. These fagopyrins have an asymmetric substitution with a methyl group and a proton at the R3 and R4 position.

Transfer of the OHO hydrogen bond in the “peri” region to the piperidine or pyrrolidine results in the formation of an OHN hydrogen bond (structures 3, 9, 15, 21, 27, and 33) and causes little change in the angles between the A–C and A–F planes. Formation of another OHN hydrogen bond in molecules 5, 11, 17, 23, 29, and 35 causes more significant changes in the polycyclic system. The changes are visible in the angle between F–H and C–H planes, so the effect of the OHN hydrogen bonds in the “peri” region on the geometry of the fagopyrin molecule is apparent and may have a real impact on the electron structure. Additionally, breaking the OHO hydrogen bond and leaving the hydroxyl group in the “peri” region as free causes deformation of the polycyclic system (structures 4, 10, 16, 22, 28, and 34). Larger differences can be observed when two free hydroxyl groups in the molecule are present (structures 6, 12, 18, 24, 30, and 36). This arrangement of the hydroxyl groups causes strong deformation of the polycyclic system of fagopyrins, which is reflected in the high energy of these conformers. So far, two similarities of the fagopyrin molecules to the hypericin molecule can be given. These are a strong influence on the geometrical structure of substituents at the R3 and R4 positions (methyl groups) and the preferred formation of the OHO hydrogen bonds in the “peri” region formed by hydroxyl groups and carbonyl oxygen.

The structures corresponding to the energy minima (2, 8, 14, 20, 26, and 32) differ from hypericin in the “bay” region. The hydroxyl groups in the “bay” region are directed to the nitrogen atom in the piperidine and pyrrolidine substituents. In the case of hypericin, the hydroxyl groups prefer the OHO hydrogen bonding. The introduction of piperidine or pyrrolidine rings at the R1 and R2 positions favors the formation of an OHN hydrogen bond and decreasing of the fagopyrin energy to a minimum.

**Figure 6:**
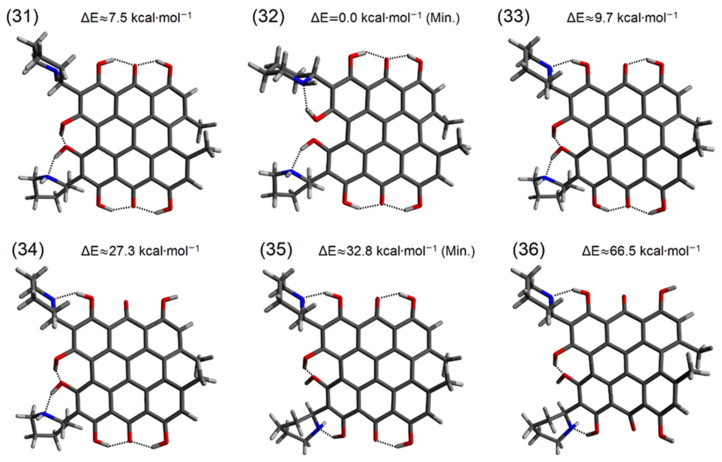
Conformers (**31**–**36**) of fagopyrin F.

Changes in the angles between the plane rings of the peripheral rings of fagopyrins result from a number of substituents and the intramolecular interactions. As fagopyrin F is the major form in the plant material [18], an analysis of hydrogen bond parameters has been performed for the structures shown in Figure 6. The length and angles of OHO and OHN hydrogen bonds are summarized in Table 2. The parameters of OHN hydrogen bonds directed to the pyrrolidine and piperidine rings are highlighted in bold. The results calculated for the fagopyrin F conformers have been compared with hypericin. The letters in parentheses in Table 2 identify the hydrogen bond location described according to Scheme 2.

Structure 31 is characterized by an arrangement of substituents similar to hypericin. The length of hydrogen bonds in the “peri” region is similar to the length of analogous bonds in hypericin. In the “bay” region, the OHO bond length is shorter than in the hypericin molecule. The OHN hydrogen bond is characterized by a length of 1.5386 Å and an angle of 156.683°. The lowest energy conformer (structure 32) is characterized by “peri”-OHO hydrogen bond lengths similar to hypericin. In structure 32, two OHN hydrogen bonds are presented in the “bay” region. The bond labeled as C(F)-O-H∙∙∙∙N(R2) is characterized by length and angle similar to the OHO “peri” bonds. The bond labeled as C(A)-O-H∙∙∙N(R1) is elongated up to 1.7233 Å. Additionally, the “peri” OHN hydrogen bond in structure 33 is longer (1.7164 Å) than the “peri” OHO hydrogen bonds.

### 3.3. Aromaticity of Fagopyrin

**Figure 7:**
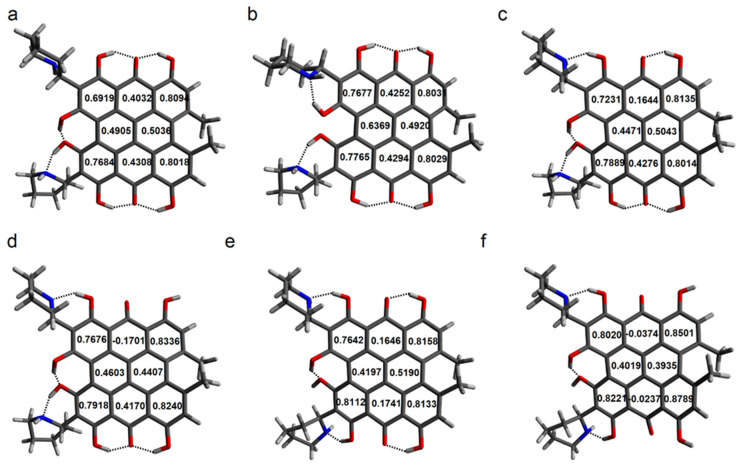
HOMA values calculated for (**a**) **31**, (**b**) **32**, (**c**) **33**, (**d**) **34**, (**e**) **35**, and (**f**) **36** fagopyrin F conformers.

The aromaticity of polycyclic compounds is related to their structure and reactivity. There are many indices describing aromaticity; however, the classical HOMA index (Harmonic Oscillator Measure of Aromaticity) is convenient for the description of aromaticity in organic compounds and relates it directly to the structure [36]. In typical aromatic compounds, the values of the HOMA index are in the range from 0 to 1, where 0 corresponds to a non-aromatic ring, and the value of 1 corresponds to a fully delocalized benzene structure, and only in special cases, the HOMA value can exceed the 0–1 range. Figure 7 shows the HOMA values calculated for the rings of the fagopyrin F conformers. The rings are marked according to Scheme 2. For hypericin, the HOMA values for particular rings are: A—0.7186, B—0.3937, C—0.8054, D—0.4712, E—0.5138, F—0.7863, G—0.4010, and H—0.7979. For the fagopyrin F structures, the peripheral rings A, C, F, and H show the highest HOMA value indicating the aromatic character of the ring. The aromaticity of the rings D and E is about 0.5. The HOMA value for rings B and G is the most variable, and for the structures 34 and 36, it is negative. Structure 31 is characterized by a hypericin-like arrangement of the substituents. The HOMA values of rings A and F are lower relatively to the hypericin; thus, the presence of the piperidine ring decreases the aromaticity of the rings. However, the D ring in structure 31 gains aromaticity relative to the hypericin moiety. The formation of the strong OHO hydrogen bonds in the “peri” region stabilizes the polycyclic system and increases the aromaticity of the central B and G rings. In general, structure 31 shows aromaticity of the rings similar to hypericin, with the influence of piperidine substituents on the aromaticity of rings A, D, and F. The lowest energy structure 32 is characterized by the formation of two OHN hydrogen bonds to the piperidine substituent in the “bay” region. This arrangement increases the aromaticity of the A, D, and F ring. Structure 33 is characterized by the breaking of the strong OHO bond in the “peri” region and the formation of an OHN hydrogen bond to the piperidine ring. Such conformation causes an increase in the energy of the system, an increase in the HOMA value of the F ring, and a decrease in the HOMA value of the B ring up to 0.1644. Breaking of another OHO bond in the “peri” region (structure 34) deepens the loss of aromaticity of the B ring. The HOMA parameter below zero indicates a complete loss of aromaticity of the ring. In structures 35 and 36, two OHN bonds in the “peri” region are present and such conformation of the hydroxyl groups causes an increase in the HOMA value in the A and F ring with significant aromaticity decreasing in the B and G ring. These changes cause increasing the total energy of the molecular system (Figure 6).

### 3.4. Analysis of Intramolecular Interactions in Fagopyrin Derivatives

**Figure 8:**
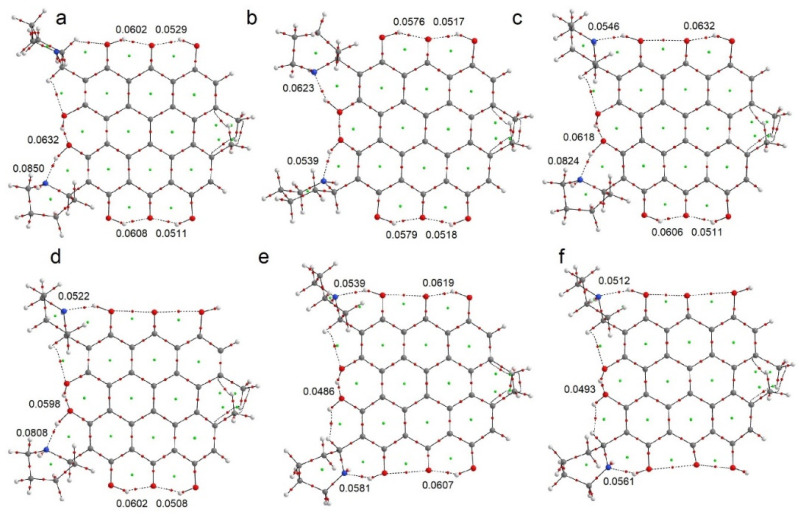
QTAIM plots for (**a**) **31**, (**b**) **32**, (**c**) **33**, (**d**) **34**, (**e**) **35**, and (**f**) **36** fagopyrin F conformers. Green points correspond to RCPs. Red points indicate BCPs.

Changes in aromaticity must be related to the changes in electron density of the polycyclic system. To describe the possible intermolecular interactions and arrangement of the electron density, the QTAIM [37] (Quantum Theory of Atoms in Molecules) analysis for the fagopyrin F conformers has been performed. In the frame of the QTAIM theory, a molecule consists of maximum, minimum, and saddle points of the electron density ρ(r). The saddle point indicates bond-critical points (BCPs) or ring-critical points (RCPs). The points representing the maximum electron density correspond to atoms. Figure 8 shows QTAIM graphs of fagopyrin F conformers. The structure of fagopyrin F is characterized by the presence of numerous substituents in a polycyclic system. Such structure allows for the occurrence of numerous intramolecular interactions of diverse nature [38]. QTAIM analysis confirms the presence of the hydrogen bond interactions in the “peri” region of fagopyrin F. Hydroxyl groups directed to carbonyl oxygen form a stable moiety as in the case of hypericin and sennidin [13,14]. QTAIM analysis indicates that OHN interaction can be formed in both the “peri” and “bay” region. The strong OHO hydrogen bonds in the anthrone moiety can be broken and replaced by weaker OHN hydrogen bonds. The electron density values at the bond critical points ρ(r) presented in Figure 8 reflect the strength of the OHO and OHN hydrogen bonds. The structures 33, 34, 35, and 36 show the formation of OHN hydrogen bonds characterized by lower values of ρ relative to the OHO hydrogen bonds in the “peri” region. However, conformer 32 (b) is characterized by the formation of OHN hydrogen bonds in the “bay” region. These bonds are characterized by a similar value of ρ(r) relative to the strong OHO hydrogen bonds in the “peri” region. This conformer shows the lowest energy; thus, the formation of strong OHN hydrogen bonds to the piperidine substituents stabilizes the anthrone system. The proximity of the piperidine ring affects the adjacent hydroxyl groups even if they do not form a direct bond. In addition, the close position of the methyl groups also introduces intermolecular interactions.

**Figure 9:**
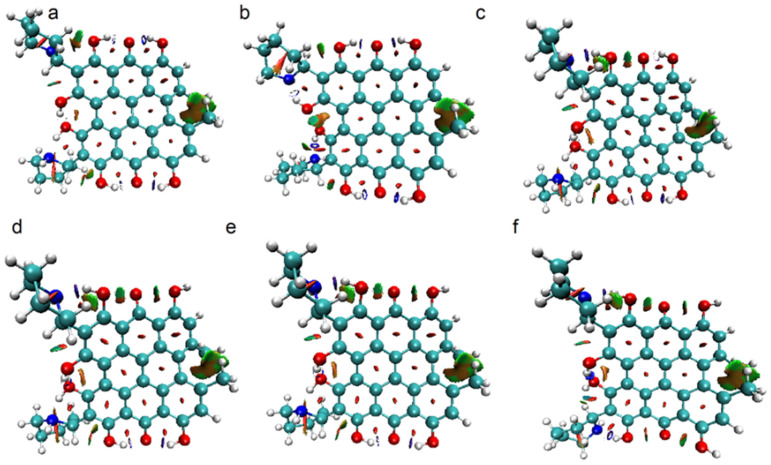
NCI plots for (**a**) **31**, (**b**) **32**, (**c**) **33**, (**d**) **34**, (**e**) **35**, and (**f**) **36** fagopyrin F conformers. Colors used for isosurfaces are: red for repulsive, green for dispersive, and blue for attractive interactions.

To confirm the presence of the interactions, the non-covalent interactions [32] (NCI) analysis was performed. Figure 9 shows the conformers of fagopyrin F, showing multiple intramolecular interactions. The blue isosurfaces in the “peri” and “bay” moiety confirm the presence of strong hydrogen bonds in the fagopyrin F molecule. Conformer 31 (a) shows similarity to hypericin in the formation of strong OHO hydrogen bonds. The lowest energy conformer 32 (b) confirms the formation of OHN hydrogen bonds in the “bay” region. Structure 33 (c) confirms the possibility of breaking the strong OHO hydrogen bond in favor of OHN hydrogen bond formation with the nitrogen atom of the piperidine ring. The interactions between the methyl groups can be described as dispersive.

### 3.5. UV-VIS Spectra of Fagopyrin Conformers

**Figure 10:**
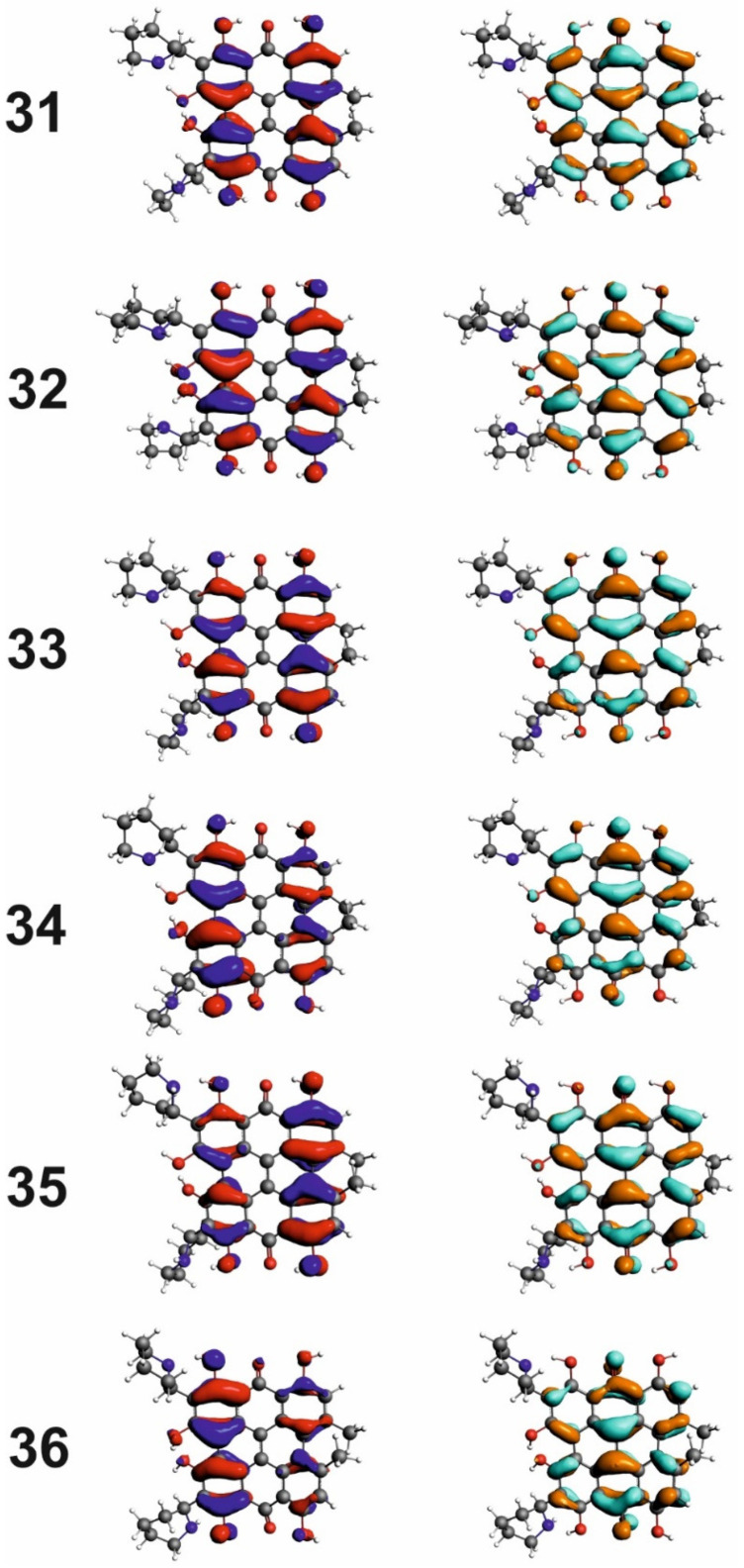
HOMO and LUMO orbitals for the fagopyrin F conformers **31**, **32**, **33**, **34**, **35**, and **36**.

Different conformation of the investigated fagopyrins is reflected in their electron structures. In Figure 10 are presented the HOMO and LUMO orbitals for the conformers of fagopyrin F—the most popular in the plant material. For other fagopyrins, the HOMO and LUMO orbitals are collected in Supplementary Materials. It is characteristic that for all conformers, the HOMO orbital is located mainly on the outer A, C, F, and H rings and on the oxygen atoms of the hydroxyl group. Only for conformers 34 and 36, the HOMO orbital is more concentrated on rings A and F than on C and H. The transfer of electrons to the LUMO orbital is connected with the shifting of electrons to the B and G rings, the oxygen of the carbonyl group, and outer bonds of the A, C, F, and H rings. Since the arrangement of the HOMO and LUMO orbitals is similar for all conformers, the HOMO–LUMO gap energy is also similar. This is persistent for all the analyzed fagopyrins. In Table 3 are collected the HOMO–LUMO gap energies for all the analyzed fagopyrins.

**Figure 11:**
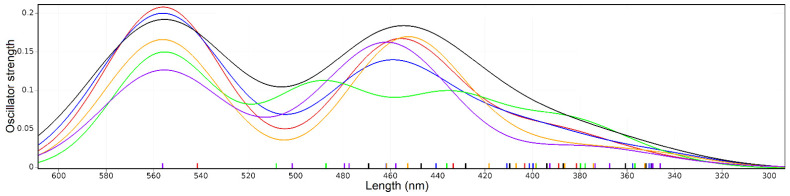
Theoretical excitation spectra for fagopyrin F conformers. Structure **31**—red, **32**—blue, **33**—black, **34**—green, **35**—orange, and **36**—purple.

**Figure 12:**
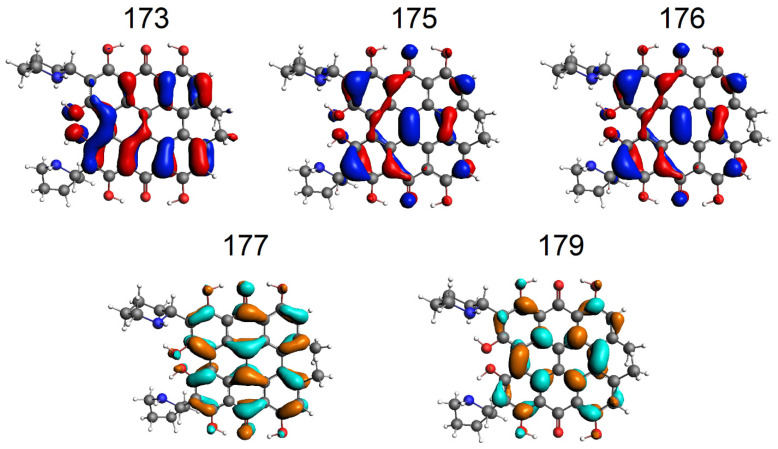
The shape of the orbitals for structure **32** (the lowest energy structure of fagopyrin F). 176—HOMO and 177—LUMO orbitals.

The UV spectra for fagopyrin F shown in Figure 11 are characterized by the presence of two intense bands. For the conformers 31, 32, and 33, the most intensive band shifts from 556 nm to 552 nm. The second intensive band is located at 457, 455, and 459 nm. For conformer 34, except for the most intensive band at 546 nm, two bands with similar intensity at 477 and 427 nm are present. The last band at 427 nm is visible in the UV-VIS spectra of 31, 32, 34, and 36 conformers; however, it is significantly lower compared to other bands. For conformers 31, 32, 33, and 34, the most intensive band is related to HOMO–LUMO transition. For conformer 35, this band is shifted to 539 nm, for conformer 36 to 503 nm, and the intensity of this band is lower than the bands at 437 and 421, respectively. For the conformers 35 and 36, transitions from lower energy orbitals are more intense than for the HOMO–LUMO transition. The electron transition participating in the bands for fagopyrin F conformers are collected in Table 4. The shape of the orbitals involved in the electron transitions in structure 32 (the lowest energy structure of fagopyrin F) is shown in Figure 12. The shape of the orbitals involved in UV-VIS transitions for fagopyrin F conformers except the presented in the text (Figure 12) is shown in Supplementary Materials.

A comparison of calculated fagopyrin F and experimental spectra [19] for the plant material suggests that in the plant material, many fagopyrin structures may be present. It is not clear which version of fagopyrin in the experimental spectra was registered; however, in the experimental spectra, the most intensive theoretically calculated bands are visible.

## 4. Conclusions

Theoretical calculations can provide information on the molecular structure when the structure is unknown, which is often the case with plant material. Fagopyrin compounds may exist as conformers characterized by a different energy. The presence of the piperidine and pyrrolidine ring in fagopyrin introduces novel intramolecular interactions compared to the double anthrone molecules. Fagopyrin A–F structures are characterized by the presence of a number of substituents and strong hydrogen bonds in the anthrone moiety. Although the OHO hydrogen bonds in the anthrone moiety are characterized as very strong, both the OHO and OHN hydrogen bonds may exist in the fagopyrin A–F structure. It is possible to break the strong OHO hydrogen bonds in the anthrone moiety in favor of interactions with the nitrogen atom in piperidine or pyrrolidine substituent. Changes in the molecular geometry are related to the changes in the orbital localization, which is reflected in the UV–VIS spectra of fagopyrin conformers.

## Figures and Tables

**Table 1:** Angles between the ring planes in fagopyrin and hypericin conformers. σ = 0.001 [deg.].

Compound	Structure	Plane/Angle [Deg.]			
		A–C	F–H	A–F	C–H
Fagopyrin A	1	13.695	12.708	23.717	30.165
	2	10.787	10.001	23.965	29.275
	3	16.005	13.476	25.420	30.763
	4	18.505	15.241	27.456	31.058
	5	16.694	14.646	30.072	30.773
	6	20.601	20.002	28.554	33.411
Fagopyrin B	7	12.227	11.517	23.245	4.320
	8	11.303	11.437	23.632	3.099
	9	17.136	11.257	26.050	5.012
	10	23.343	8.266	26.391	2.675
	11	17.52	15.562	30.230	4.862
	12	20.611	20.796	33.032	5.543
Fagopyrin C	13	10.728	12.357	24.436	19.975
	14	8.323	10.393	24.598	18.545
	15	13.846	12.925	25.928	20.427
	16	17.886	13.166	27.414	18.563
	17	15.129	18.324	30.626	20.972
	18	17.561	20.219	33.223	20.238
Fagopyrin D	19	10.778	12.366	24.422	19.978
	20	8.305	10.341	24.604	18.515
	21	13.847	12.918	25.848	20.431
	22	17.800	13.066	27.297	18.533
	23	14.637	16.649	30.708	20.473
	24	17.558	20.262	33.338	20.209
Fagopyrin E	25	13.469	12.696	24.195	30.168
	26	10.838	10.654	24.508	29.384
	27	15.981	13.491	25.502	30.778
	28	18.502	15.112	27.347	30.987
	29	16.798	14.604	30.088	30.787
	30	21.120	22.148	27.824	33.555
Fagopyrin F	31	13.482	12.721	24.089	30.172
	32	10.805	10.638	24.461	29.384
	33	16.011	13.53	25.430	30.789
	34	18.581	15.220	27.322	30.993
	35	17.361	16.870	30.244	31.195
	36	21.112	21.813	27.898	33.568
Hypericin	-	13.334	12.363	23.188	30.095

**Table 2:** Hydrogen bonds in possible fagopyrin F conformers. σ = 0.0001 [Å], σ = 0.001 [deg.].

Structure	Hydrogen Bond Location	H∙∙∙O/H∙∙∙N [Å]	O∙∙∙O/O∙∙∙N [Å]	OHO/OHN [deg.]
31	C(A)-O-H∙∙∙O=C(B)	1.6047	2.5133	149.530
	C(C)-O-H∙∙∙O=C(B)	1.6583	2.5485	147.083
	C(F)-O-H∙∙∙O=C(G)	1.6022	2.5125	149.135
	C(H)-O-H∙∙∙O=C(G)	1.6721	2.5591	146.198
	C(A)-O-H∙∙∙O-C(F)	1.5751	2.5155	158.577
	**C(F)-O-H∙∙∙N(R2)**	**1.5386**	**2.5278**	**156.683**
32	C(A)-O-H∙∙∙O=C(B)	1.6220	2.5319	149.412
	C(C)-O-H∙∙∙O=C(B)	1.6667	2.5580	147.383
	C(F)-O-H∙∙∙O=C(G)	1.6244	2.5338	149.346
	C(H)-O-H∙∙∙O=C(G)	1.6677	2.5589	147.388
	**C(A)-O-H∙∙∙N(R1)**	**1.7233**	**2.6379**	**150.007**
	**C(F)-O-H∙∙∙N(R2)**	**1.6676**	**2.6012**	**152.071**
33	**C(A)-O-H∙∙∙N(R1)**	**1.7164**	**2.6276**	**149.868**
	C(C)-O-H∙∙∙O=C(B)	1.5936	2.5098	149.406
	C(F)-O-H∙∙∙O=C(G)	1.6035	2.5143	149.243
	C(H)-O-H∙∙∙O=C(G)	1.6720	2.5597	147.032
	C(A)-O-H∙∙∙O-C(F)	1.5849	2.5207	157.420
	**C(F)-O-H∙∙∙N(R2)**	**1.5508**	**2.5354**	**156.626**
34	**C(A)-O-H∙∙∙N(R1)**	**1.7350**	**2.6378**	**148.993**
	C(F)-O-H∙∙∙O=C(G)	1.6062	2.5164	149.237
	C(H)-O-H∙∙∙O=C(G)	1.6750	2.5627	147.055
	C(A)-O-H∙∙∙O-C(F)	1.5977	2.5319	157.421
	**C(F)-O-H∙∙∙N(R2)**	**1.5590**	**2.5402**	**156.438**
35	**C(A)-O-H∙∙∙N(R1)**	**1.7220**	**2.6316**	**149.766**
	C(C)-O-H∙∙∙O=C(B)	1.6017	2.5156	149.256
	**C(F)-O-H∙∙∙N(R2)**	**1.6940**	**2.6188**	**152.744**
	C(H)-O-H∙∙∙O=C(G)	1.6095	2.5203	149.017
	C(A)-O-H∙∙∙O-C(F)	1.6717	2.5572	149.732
36	**C(A)-O-H∙∙∙N(R1)**	**1.7438**	**2.6437**	**148.744**
	**C(F)-O-H∙∙∙N(R2)**	**1.7087**	**2.6243**	**151.524**
	C(A)-O-H∙∙∙O-C(F)	1.6658	2.5536	150.072
Hypericin	C(A)-O-H∙∙∙O=C(B)	1.6499	2.5422	147.330
	C(C)-O-H∙∙∙O=C(B)	1.6659	2.5548	146.940
	C(F)-O-H∙∙∙O=C(G)	1.6400	2.5351	147.385
	C(H)-O-H∙∙∙O=C(G)	1.6750	2.5606	146.668
	C(A)-O-H∙∙∙O-C(F)	1.6670	2.5605	151.168

**Table 3:** HOMO–LUMO gap energy for calculated fagopyrin structures.

Structure	HOMO–LUMO Gap [kcal∙mol^−1^]
1	58.4
2	58.4
3	59.2
4	61.0
5	61.0
6	66.5
7	59.7
8	59.9
9	60.2
10	61.7
11	62.7
12	67.9
13	59.1
14	59.3
15	60.0
16	61.6
17	62.2
18	67.3
19	59.0
20	59.3
21	60.0
22	61.6
23	62.1
24	67.1
25	58.4
26	58.5
27	59.5
28	61.1
29	61.1
30	67.1
31	58.4
32	58.5
33	59.4
34	61.1
35	61.2
36	66.9

**Table 4:** The electron transition contribution and orbitals for fagopyrin F conformers.

Structure	Band [nm]	Orbital Transition	Transition Contribution [%]
31	556	176(HOMO) → 177(LUMO)	95.7
		175 → 177	1.3
	457	174 → 177	90.3
		172 → 177	3.0
		176 → 179	2.7
		173 → 177	1.3
32	552	176(HOMO) → 177(LUMO)	96.7
	455	175 → 177	91.2
		173 → 177	3.6
		176 → 179	3.1
33	552	176(HOMO) → 177(LUMO)	97.1
	459	175 → 177	86.7
		174 → 177	7.7
		176 → 179	1.5
		173 → 177	1.4
	436	174 → 177	87.1
		175 → 177	7.0
		176 → 179	2.2
		173 → 177	1.2
34	546	176(HOMO) → 177(LUMO)	96.0
	477	175 → 177	70.7
		174 → 177	17.3
		173 → 177	6.8
		176 → 179	1.1
	427	173 → 177	82.6
		174 → 177	10.2
		176 → 179	1.9
		172 → 177	1.2
		175 → 177	1.1
35	539	176(HOMO) → 177(LUMO)	97.3
	437	175 → 177	74.5
		174 → 177	17.2
		176 → 179	2.7
		172 → 177	1.8
36	503	176(HOMO) → 177(LUMO)	96.8
	421	174 → 177	52.5
		175 → 177	29.4
		172 → 177	9.5
		176 → 179	3.1
		171 → 177	1.1

## Data Availability

The data presented in this study are available on request from the corresponding author.

## Supplementary Materials

The following supporting information can be downloaded at: https://www.mdpi.com/article/10.3390/molecules27123689/s1. Figure S1. HOMO (left) and LUMO (right) orbitals for the 1, 2, 3, 4, 5, and 6 Fagopyrin A conformers. Figure S2. HOMO (left) and LUMO (right) orbitals for the 7, 8, 9, 10, 11, and 12 Fagopyrin B conformers. Figure S3. HOMO (left) and LUMO (right) orbitals for the 13, 14, 15, 16, and 17 Fagopyrin C conformers. Figure S4. HOMO (left) and LUMO (right) orbitals for the 19, 20, 21, 22, 23, and 24 Fagopyrin D conformers. Figure S5. HOMO (left) and LUMO (right) orbitals for the 25, 26, 27, 28, 29, and 30 Fagopyrin E conformers. Figure S6. The shape of the orbitals for structure 31 (Fagopyrin F). 176—HOMO and 177—LUMO orbitals. Figure S7. The shape of the orbitals for structure 33 (Fagopyrin F). 176—HOMO and 177—LUMO orbitals. Figure S8. The shape of the orbitals for structure 34 (Fagopyrin F). 176—HOMO and 177—LUMO orbitals. Figure S9. The shape of the orbitals for structure 35 (Fagopyrin F). 176—HOMO and 177—LUMO orbitals. Figure S10. The shape of the orbitals for structure 36 (Fagopyrin F). 176—HOMO and 177—LUMO orbitals.

## Sample Availability

Samples of the compounds are not available from the authors.

## References

[1] Babu S., Yadav G.S., Singh R., Avasthe R.K., Das A., Mohapatra K.P., Tahashildar M., Kumar K., Prabha M., Thoithoi Devi M. (2018). Production technology and multifarious uses of buckwheat (*Fagopyrum* spp.): A review. Indian J. Agron..

[2] Sytar O., Brestic M., Zivcak M., Phan Tran L.-S. (2016). The Contribution of Buckwheat Genetic Resources to Health and Dietary Diversity. Curr. Genom..

[3] Giupponi L., Borgonovo G., Panseri S., Giorgi A. (2019). Multidisciplinary study of a little known landrace of Fagopyrum tataricum Gaertn. of Valtellina (Italian Alps). Genet. Resour. Crop Evol..

[4] Ožbolt L., Kreft S., Kreft I., Germ M., Stibilj V. (2008). Distribution of selenium and phenolics in buckwheat plants grown from seeds soaked in Se solution and under different levels of UV-B radiation. Food Chem..

[5] Habtemariam S. (2019). Antioxidant and rutin content analysis of leaves of the common buckwheat (*fagopyrum esculentum* moench) grown in the United Kingdom: A case study. Antioxidants.

[6] Dziedzic K., Górecka D., Szwengiel A., Sulewska H., Kreft I., Gujska E., Walkowiak J. (2018). The Content of Dietary Fibre and Polyphenols in Morphological Parts of Buckwheat (*Fagopyrum tataricum*). Plant Foods Hum. Nutr..

[7] Kočevar Glavač N., Stojilkovski K., Kreft S., Park C.H., Kreft I. (2017). Determination of fagopyrins, rutin, and quercetin in Tartary buckwheat products. LWT—Food Sci. Technol..

[8] Sytar O., Brestic M., Rai M. (2013). Possible ways of fagopyrin biosynthesis and production in buckwheat plants. Fitoterapia.

[9] Szymańska M., Majerz I. (2020). Geometry and electron density of phenothazines. J. Mol. Struct..

[10] Szymańska M., Majerz I. (2021). Effect of substitution of hydrogen atoms in the molecules of anthrone and anthraquinone. Molecules.

[11] Edim M.M., Enudi O.C., Asuquo B.B., Louis H., Bisong E.A., Agwupuye J.A., Chioma A.G., Odey J.O., Joseph I., Bassey F.I. (2021). Aromaticity indices, electronic structural properties, and fuzzy atomic space investigations of naphthalene and its aza-derivatives. Heliyon.

[12] Galinari C.B., Biachi T.D.P., Gonçalves R.S., Cesar G.B., Bergmann E.V., Malacarne L.C., Kioshima Cotica É.S., Bonfim-Mendonça P.D.S., Svidzinski T.I.E. (2022). Photoactivity of hypericin: From natural product to antifungal application. Crit. Rev. Microbiol..

[13] Szymanski S., Majerz I. (2019). Aromaticity and Electron Density of Hypericin. J. Nat. Prod..

[14] Szymanski S., Majerz I. (2021). In silico studies on sennidines—Natural dianthrones from senna. Biology.

[15] Eguchi K., Anase T., Osuga H. (2009). Development of a high-performance liquid chromatography method to determine the fagopyrin content of Tartary buckwheat (*Fagopyrum tartaricum* Gaertn.) and common buckwheat (*F. esculentum* Moench). Plant Prod. Sci..

[16] Kim J., Kim S., Hwang K.T. (2021). Determination and photochemical conversion of protofagopyrins and fagopyrins in buckwheat plants. J. Food Compos. Anal..

[17] Benković E.T., Žigon D., Friedrich M., Plavec J., Kreft S. (2014). Isolation, analysis and structures of phototoxic fagopyrins from buckwheat. Food Chem..

[18] Kim J., Hwang K.T. (2020). Fagopyrins in different parts of common buckwheat (*Fagopyrum esculentum*) and Tartary buckwheat (*F. tataricum*) during growth. J. Food Compos. Anal..

[19] Kosyan A., Sytar O. (2021). Implications of fagopyrin formation in vitro by uv spectroscopic analysis. Molecules.

[20] Samel D., Donnella-Deana A., De Witte P. (1996). The effect of purified extract of Fagopyrum esculentum (buckwheat) on protein kinases involved in signal transduction pathways. Planta Med..

[21] Kim J., Kim S., Lee K., Kim R.H., Hwang K.T. (2021). Antibacterial photodynamic inactivation of fagopyrin f from tartary buckwheat (*Fagopyrum tataricum*) flower against streptococcus mutans and its biofilm. Int. J. Mol. Sci..

[22] Tavčar Benković E., Kreft S. (2015). Fagopyrins and Protofagopyrins: Detection, Analysis, and Potential Phototoxicity in Buckwheat. J. Agric. Food Chem..

[23] Lineva A., Benković E.T., Kreft S., Kienzle E. (2019). Remarkable frequency of a history of liver disease in dogs fed homemade diets with buckwheat. Tierärztliche Prax. Ausg. K Kleintiere-Heimtiere.

[24] Sytar O., Švedienė J., Ložienė K., Paškevičius A., Kosyan A., Taran N. (2016). Antifungal properties of hypericin, hypericin tetrasulphonic acid and fagopyrin on pathogenic fungi and spoilage yeasts. Pharm. Biol..

[25] Zambounis A., Sytar O., Valasiadis D., Hilioti Z. (2020). Effect of photosensitisers on growth and morphology of phytophthora citrophthora coupled with leaf bioassays in pear seedlings. Plant Prot. Sci..

[26] Pauling L. (1960). The Nature of the Chemical Bond an Introduction to Modern Structural Chemistry.

[27] Schuster P., Zundel G., Sandorfy C. (1975). The Hydrogen Bond, II: Structure and Spectroscopy.

[28] Gilli G., Gilli P. (2009). The Nature of the Hydrogen Bond: Outline of a Comprehensive Hydrogen Bond Theory.

[29] Frisch G.W., Schlegel H.B., Scuseria G.E., Robb M.A., Cheeseman J.R., Scalmani G., Barone V., Petersson G.A., Nakatsuji H., Li X. (2016). Gaussian 16, Rev. A.03.

[30] Grimme S., Antony J., Ehrlich S., Krieg H. (2010). A consistent and accurate ab initio parametrization of density functional dispersion correction (DFT-D) for the 94 elements H-Pu. J. Chem. Phys..

[31] Keith T.A. (2019). AIMALL.

[32] Johnson E.R., Keinan S., Mori-Sánchez P., Contreras-García J., Cohen A.J., Yang W. (2010). Revealing noncovalent interactions. J. Am. Chem. Soc..

[33] Lu T., Chen F. (2012). Multiwfn: A multifunctional wavefunction analyzer. J. Comput. Chem..

[34] Humphrey W., Dalke A., Schulten K. (1996). VMD: Visual molecular dynamics. J. Mol. Graph..

[35] te Velde G., Bickelhaupt F.M., Baerends E.J., Fonseca Guerra C., van Gisbergen S.J.A., Snijders J.G., Ziegler T. (2001). Chemistry with ADF. J. Comput. Chem..

[36] Krygowski T.M. (1993). Crystallographic Studies of Inter- and Intramolecular Interactions Reflected in Aromatic Character of π-Electron Systems. J. Chem. Inf. Comput. Sci..

[37] Richard F. (1990). Bader: Atoms in Molecules (A Quantum Theory).

[38] Bader R.F.W. (2009). Bond paths are not chemical bonds. J. Phys. Chem. A.

