# Supplementary material for: Theoretical Studies on the Structure and Intramolecular Interactions of Fagopyrins—Natural Photosensitizers of Fagopyrum
Source: Molecules. 2022 Jun 8;27(12):3689. doi: 10.3390/molecules27123689 (PMC9230917; doi:10.3390/molecules27123689)
Supplement: Supplementary file 1 [file molecules-27-03689-s001.zip › molecules-1720342-supplementary.pdf]

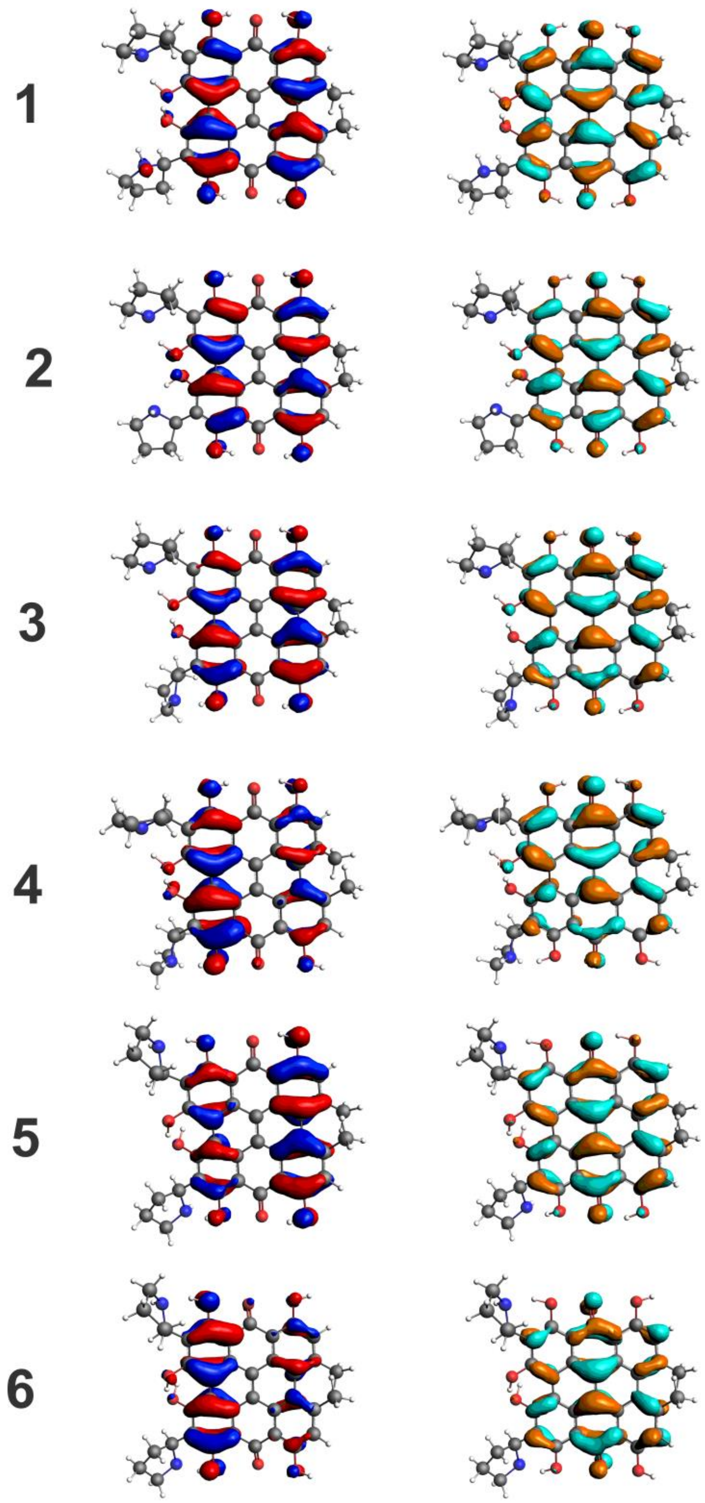

Figure S1. HOMO (left) and LUMO (right) orbitals for the 1, 2, 3, 4, 5, 6 Fagopyrin A conformers.

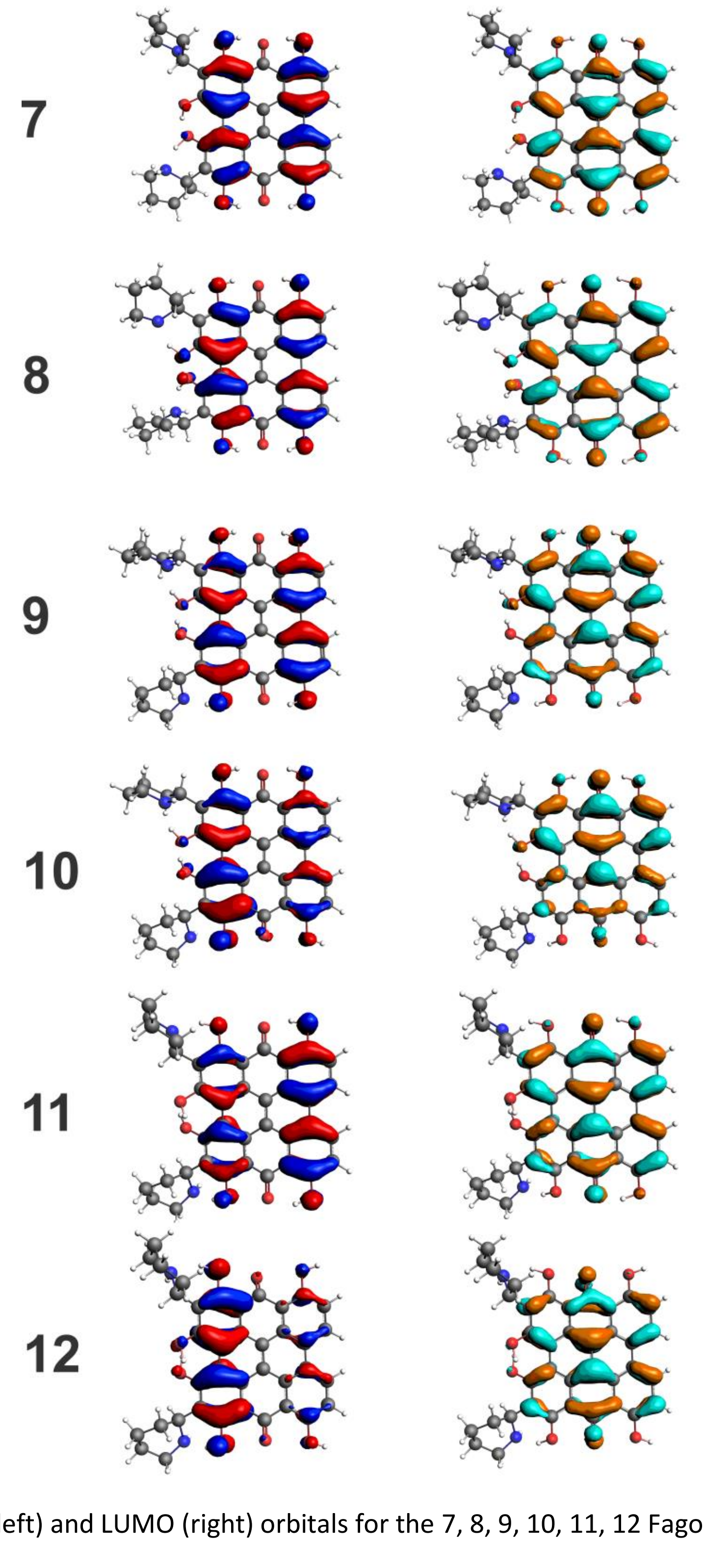

Figure S2. HOMO (left) and LUMO (right) orbitals for the 7, 8, 9, 10, 11, 12 Fagopyrin B conformers.

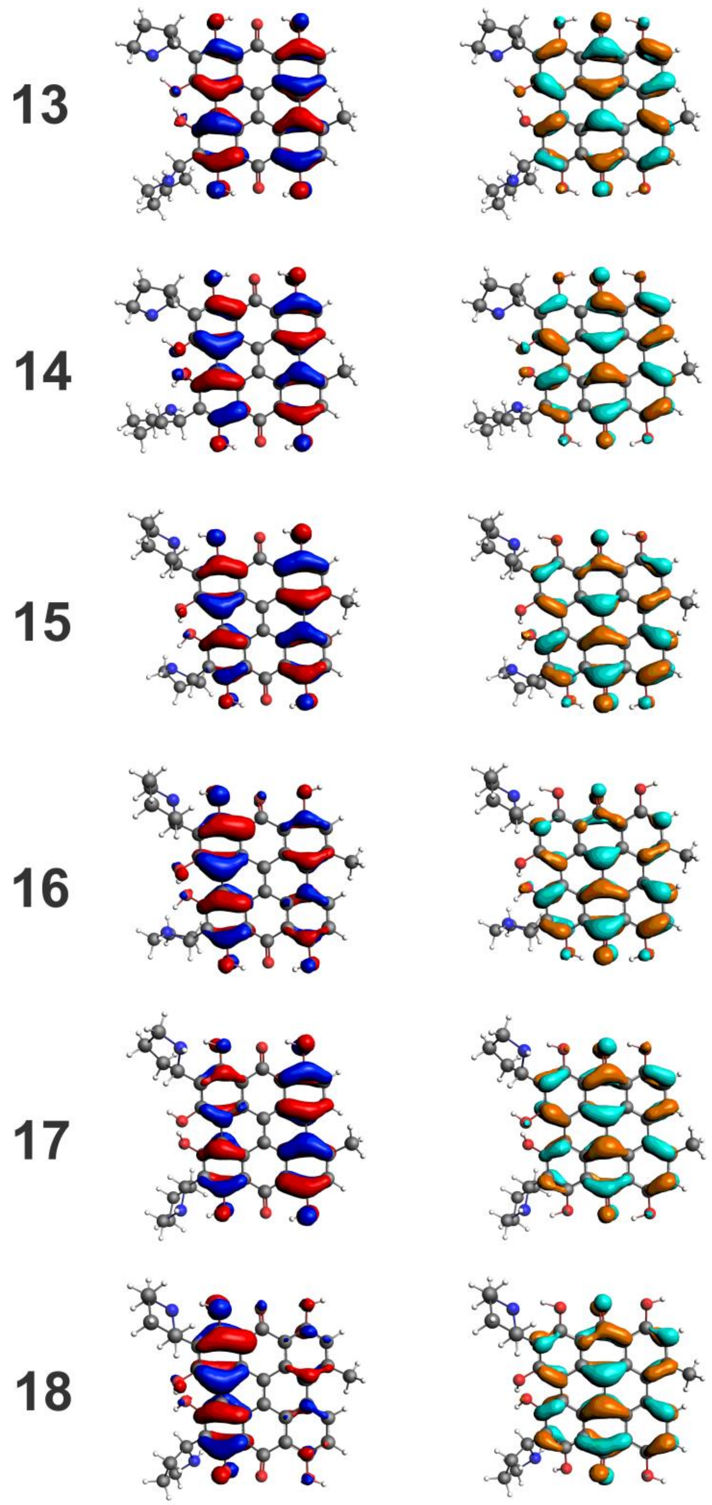

Figure S3. HOMO (left) and LUMO (right) orbitals for the 13, 14, 15, 16, 17 Fagopyrin C conformers.

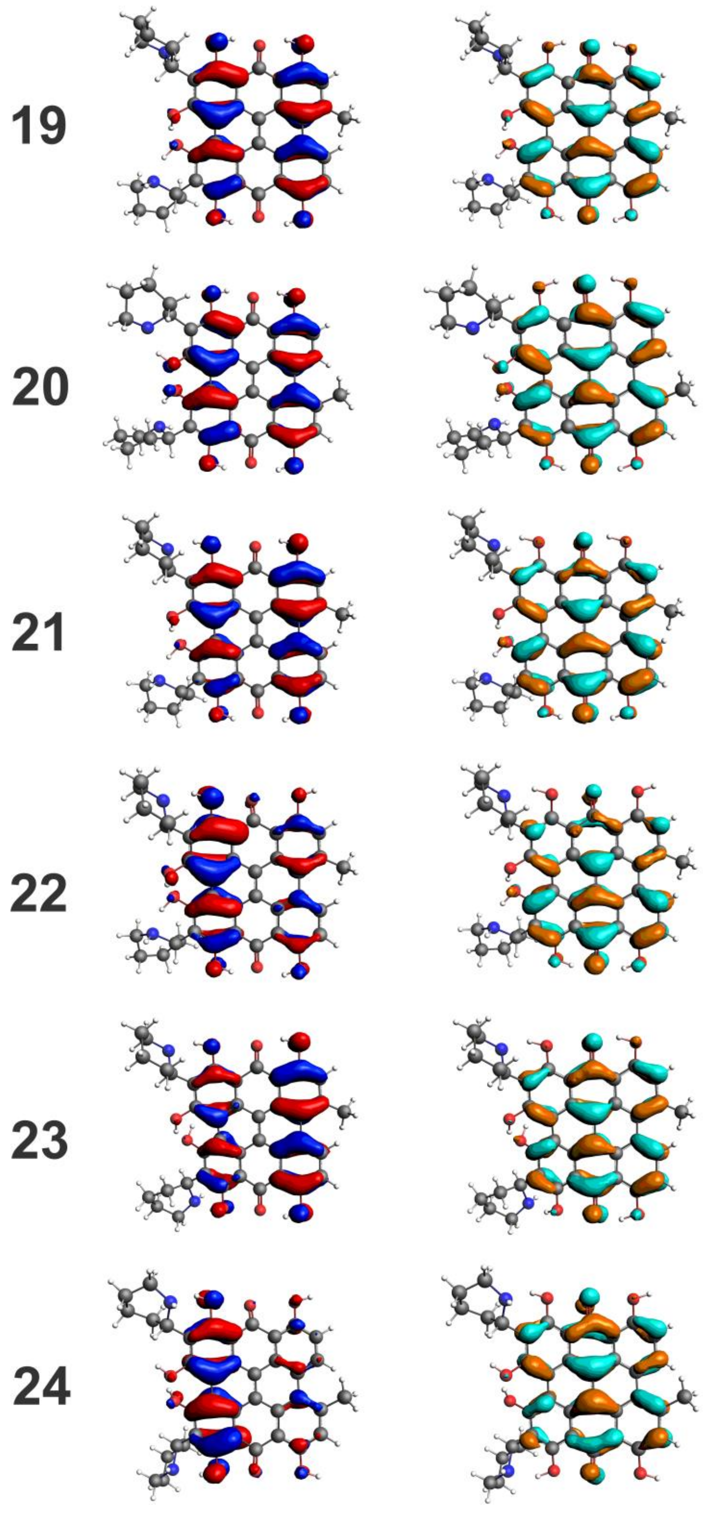

Figure S4. HOMO (left) and LUMO (right) orbitals for the 19, 20, 21, 22, 23, 24 Fagopyrin D conformers.

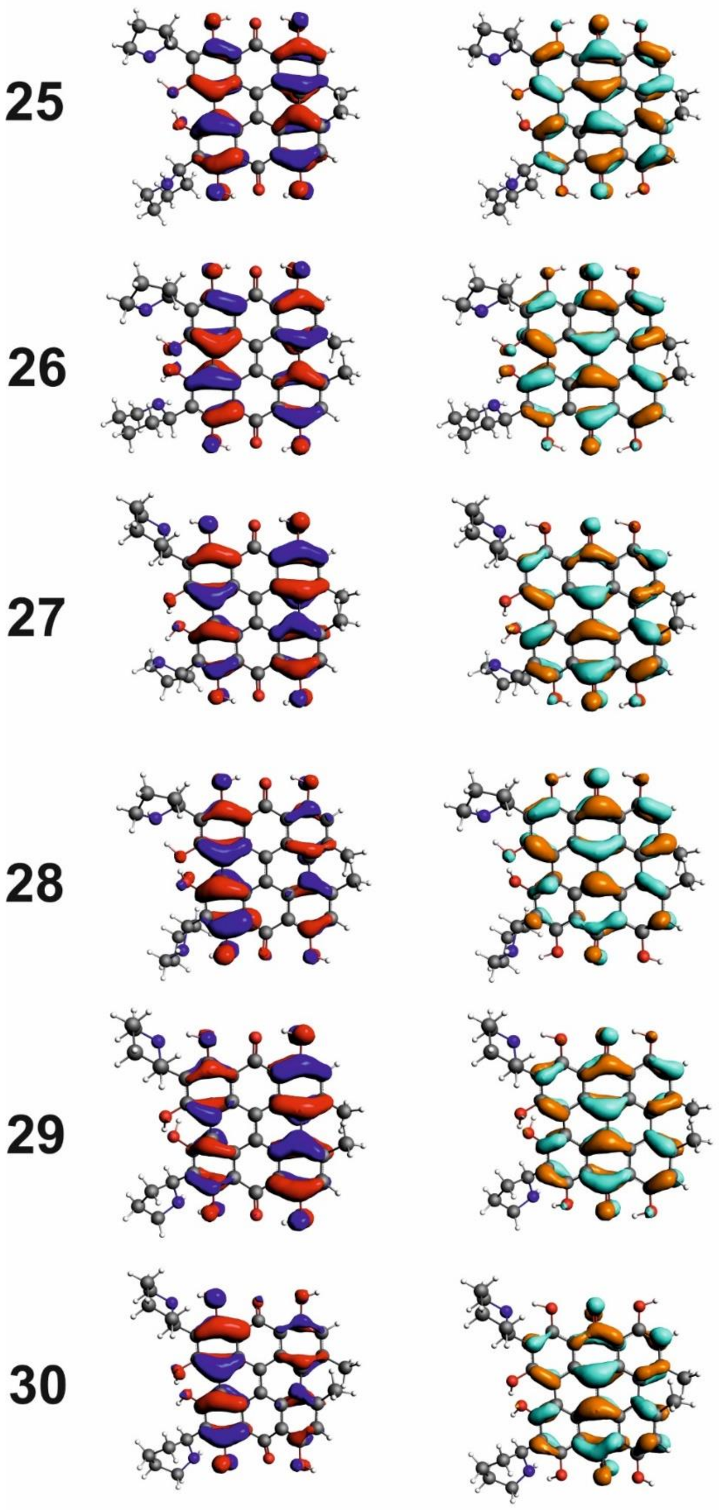

Figure S5. HOMO (left) and LUMO (right) orbitals for the 25, 26, 27, 28, 29, 30 Fagopyrin E conformers.

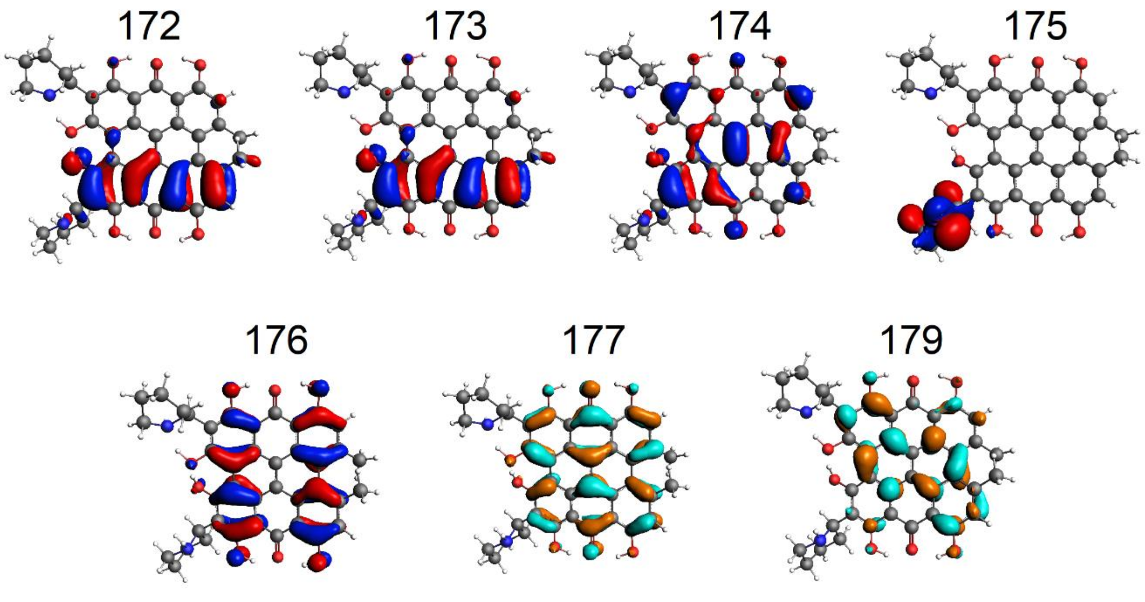

Figure S6. The shape of the orbitals for structure 31 (Fagopyrin F). 176 – HOMO and 177 – LUMO orbitals.

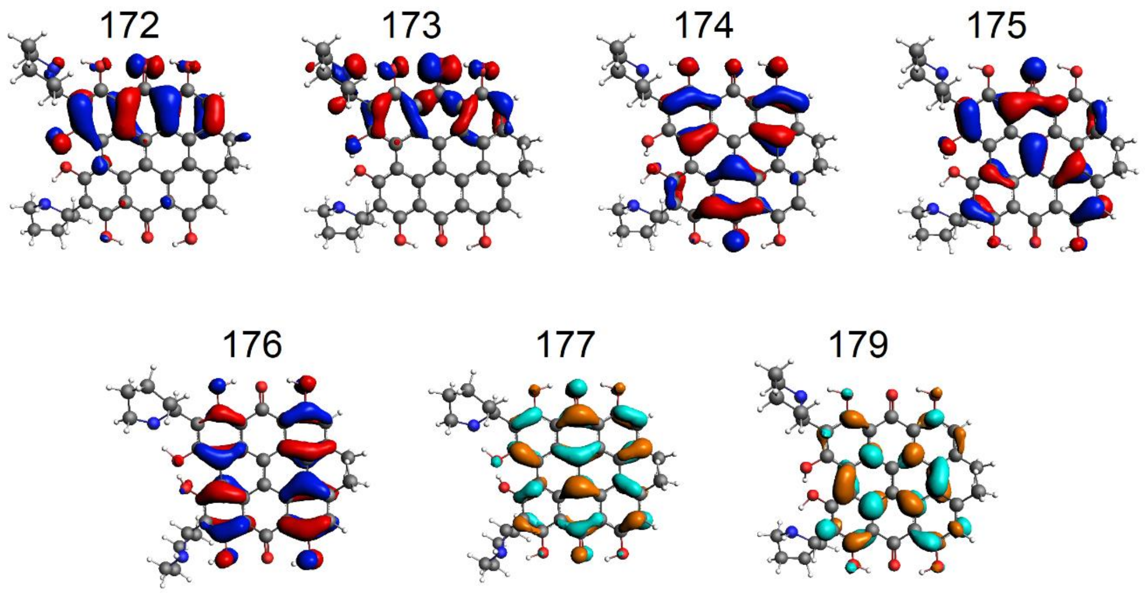

Figure S7. The shape of the orbitals for structure 33 (Fagopyrin F). 176 – HOMO and 177 – LUMO orbitals.

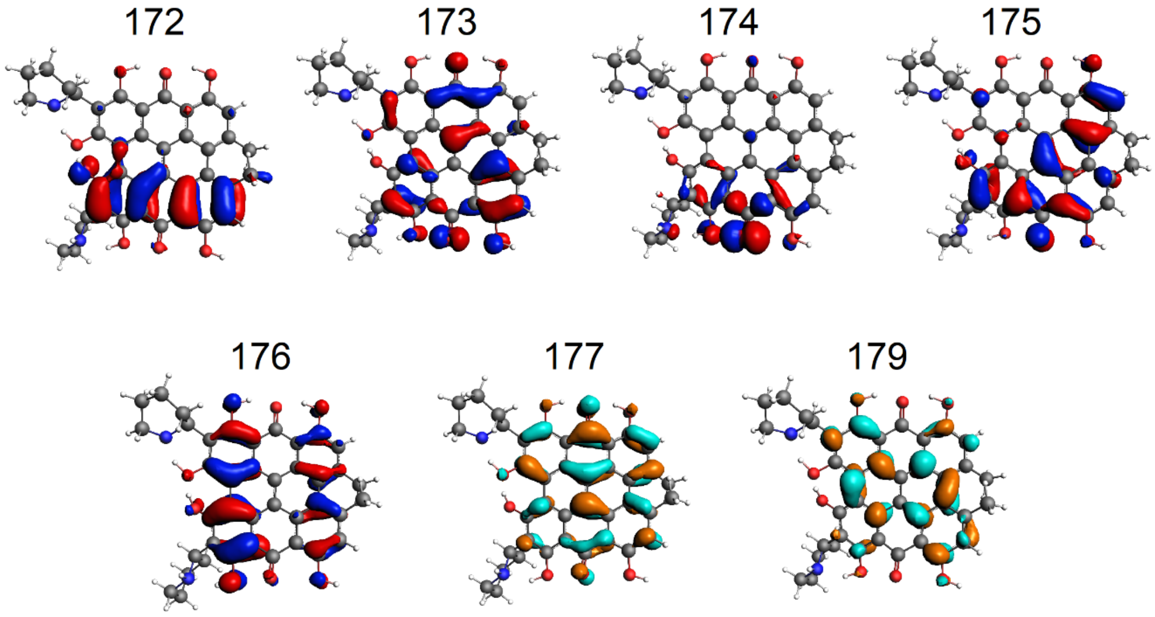

Figure S8. The shape of the orbitals for structure 34 (Fagopyrin F). 176 – HOMO and 177 – LUMO orbitals.

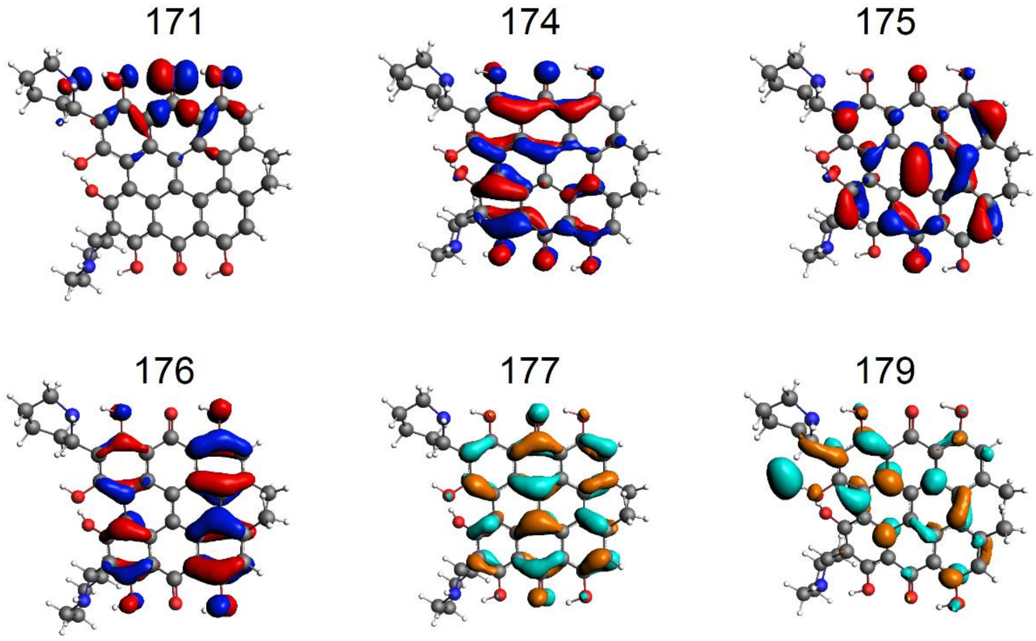

Figure S9. The shape of the orbitals for structure 35 (Fagopyrin F). 176 – HOMO and 177 – LUMO orbitals.

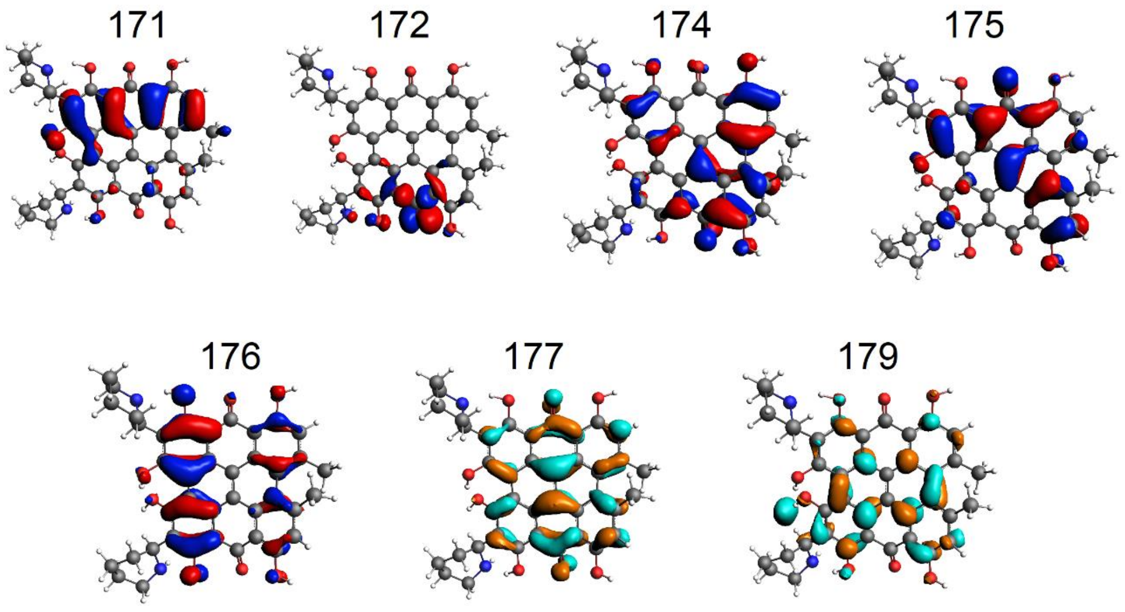

Figure S10. The shape of the orbitals for structure 36 (Fagopyrin F). 176 – HOMO and 177 – LUMO orbitals.
